# Antimicrobial and antioxidant activities of *Cortex Magnoliae Officinalis *and some other medicinal plants commonly used in South-East Asia

**DOI:** 10.1186/1749-8546-3-15

**Published:** 2008-11-28

**Authors:** Lai Wah Chan, Emily LC Cheah, Constance LL Saw, Wanyu Weng, Paul WS Heng

**Affiliations:** 1 Department of Pharmacy, Faculty of Science, National University of Singapore, 18 Science Drive 4, Singapore 117543; 2 Center for Cancer Prevention Research, Department of Pharmaceutics, Ernest Mario School of Pharmacy, Rutgers, State University of New Jersey, 160 Frelinghuysen Road, Piscataway, New Jersey 08854, USA

## Abstract

**Background:**

Eight medicinal plants were tested for their antimicrobial and antioxidant activities. Different extraction methods were also tested for their effects on the bioactivities of the medicinal plants.

**Methods:**

Eight plants, namely *Herba Polygonis Hydropiperis *(*Laliaocao*), *Folium Murraya Koenigii *(*Jialiye*), *Rhizoma Arachis Hypogea *(*Huashenggen*), *Herba Houttuyniae *(*Yuxingcao*), *Epipremnum pinnatum *(*Pashulong*), *Rhizoma Typhonium Flagelliforme *(*Laoshuyu*), *Cortex Magnoliae Officinalis *(*Houpo*) and *Rhizoma Imperatae *(*Baimaogen*) were investigated for their potential antimicrobial and antioxidant properties.

**Results:**

Extracts of *Cortex Magnoliae Officinalis *had the strongest activities against *M. Smegmatis*, *C. albicans*, *B. subtilis *and *S. aureus*. Boiled extracts of *Cortex Magnoliae Officinalis*, *Folium Murraya Koenigii, Herba Polygonis Hydropiperis *and *Herba Houttuyniae *demonstrated greater antioxidant activities than other tested medicinal plants.

**Conclusion:**

Among the eight tested medicinal plants, *Cortex Magnoliae Officinalis *showed the highest antimicrobial and antioxidant activities. Different methods of extraction yield different spectra of bioactivities.

## Background

Some medicinal plants used in traditional Chinese medicine are effective in treating various ailments caused by bacterial and oxidative stress. As new drug-resistant bacteria strains emerge, especially methicillin-resistant *Staphylococcus aureus *and vancomycin-resistant enterococci, new drugs or adjuvants have been actively searched in medicinal plants [1-3]. New antioxidants such as plant phenolics [4-7] are sought for general health maintenance, anti-aging and chemoprevention.

Eight medicinal plants, namely *Herba Polygonis Hydropiperis *(*Laliaocao*), *Folium Murraya Koenigii *(*Jialiye*), *Rhizoma Arachis Hypogea *(*Huashenggen*), *Herba Houttuyniae *(*Yuxingcao*), *Epipremnum pinnatum *(*Pashulong*), *Rhizoma Typhonium Flagelliforme *(*Laoshuyu*), *Cortex Magnoliae Officinalis *(*Houpo*) and *Rhizoma Imperatae *(*Baimaogen*) were tested for their potential antimicrobial and antioxidant properties. They have been long been used in treating of various infectious diseases, e.g. skin/wound infections, fever, cough and digestive ailments (Table 1, [8-33]).

**Table 1:** Ethnomedicinal uses and properties of the selected plants

**Latin pharmaceutical name/Plant scientific name/Family/Voucher specimen no**.	**Vernacular/pinyin names**	**Ethnomedicinal uses**	**Properties**
*Herba Polygonis Hydropiperis/Persicaria hydropiper *(L.)^a ^Spach/Polygonaceae/001-CS0807	Laksa plant/*Laliaocao*	Used as a condiment. Also employed as a stomachic and aphrodisiac. Externally, the crushed leaves or juice are used to treat skin conditions such as ringworms, scabies, boils, abscesses, carbuncles, ulcers or bites of snakes, dogs or insects	Antioxidant [8-10]
*Folium Murraya Koenigii/Murraya koenigii Spreng./*Rutaceae/002-CS0807	Curry leaves/*Jialiye*	Used as a condiment. Treatment of piles, inflammation, itching, fresh cuts, dysentery, vomiting, burses and dropsy	Reducing halitosis [11], antioxidant [12], antimicrobial [13], antifungal [14], antihyperglycemic and antihyperlipidemic properties [15]
Rhizoma Arachis Hypogea/Arachis hypogaea *L./Leguminosae/003-CS0807*	Groundnut/*Huashenggen*	Treatment of insomnia and strengthening of bones	Antifibrinolytic [16]
*Herba Houttuyniae/Houttuynia cordata *Thunb./Saururaceae/004-CS0807	Chinese houttuynia or chameleon plant/*Yuxingcao*	Detoxification, treatment of infection, removing toxic heat, promoting drainage of pus and urination	Anti-Severe Acute Respiratory Syndrome (SARS) [17]. Prevention of urinary infection, modulation of neutrophils and monocytes, inhibition of respiratory bacteria [18,19]. Anti-inflammatory activity [20]. Virucidal effects on herpes simplex virus type 1 and 2, influenza virus, and human immunodeficiency virus type 1 [21,22]
*Epipremnum pinnatum *(L.) Engl./Araceae/005-CS0807	Dragon tail/*Pashulong*	Detoxification, removes toxic heat, tendonitis, fractures, burns, carbuncles, sores, redness	Cytotoxicity against cancers cells [23], immuno-modulating [24]
*Rhizoma Typhonium Flagelliforme/Typhonium flagelliforme *(Lodd.) Blume/006-CS0807	Rodent tuber/*Laoshuyu*	Treatment of cough, asthma, nausea and cancers	Relieving cough, eliminating phlegm, asthmatic, analgesia, anti-inflammation, sedation and cytotoxic activities [25-28]
*Cortex Magnoliae Officinalis/Magnolia biloba *(Rehder & E. H. Wilson) Cheng/Magnoliaceae/007-CS0807	Magnolia/*Houpo*	A tonic to improve general well-being, also used to treat cough, diarrhea, allergic rhinitis and phlegm	Alleviateing menopausal symptoms [29], brochial asthma [30,31], active against *Propionibacterium acnes *and *Propionibacterium granulosum *[32], antimicrobial and cytotoxic activities [33,34]
*Rhizoma Imperatae/Imperata cylidrica *(L.) Beeuv. var. *major *(Nees) C.E. Hubb/Gramineae/008-CS0807	Lalang/*Baimaogen*	Wound-healing, diuretic, anti-inflammatory and antipyretic agents	Neuroprotective, immunostimulating effects [35]

^a^*Persicaria hydropiper *(L.) is synonymous with *Polygonum hydropiper (L.). Persicaria hydropiper *(L.) and *Persicaria odoratum *(L.) are commonly used interchangeably in literature while they are two distinct species. Efforts were made to identify the species of laksa plants used in the study. The plant was probably *Persicaria hydropiper *(L.). A specimen of the plant has been deposited in the National University of Singapore Herbarium for future reference.

The traditional method for Chinese medicine preparation is to boil the medicinal plants in water for 20 minutes to one hour. The present study aims to test the effectiveness of traditional herb preparation methods for antimicrobial and antioxidant treatments.

## Methods

### Materials

#### Selection of plants

The rationales behind the selection of these eight plants are as follows. (1) They are commonly used in Asia. (2) They have long been used as medicinal plants. (3) They are abundant in the market. (4) Their daily applications have not been documented (except *Cortex Magnoliae Officinalis* which served as a positive control for its antimicrobial activity against *S. aureus*). The fresh juices of some of the plants were traditionally used as fresh poultices to treat some skin conditions (Table 1).

#### Plant materials

*Cortex Magnoliae Officinalis *from Zhejiang, China was purchased from WHL Ginseng & Herbs (Singapore), while all other plants were purchased from a herbal vendor in Outram Park wet market in Singapore. *Cortex Magnoliae Officinalis *and *Rhizoma Imperatae *were authenticated by the Institute of Medicinal Plant Development of the Chinese Academy of Medical Sciences (China), while the rest were authenticated by the Herbarium of the Singapore Botanic Gardens (Singapore). The voucher specimens for each plant were preserved under the reference number 001-CS0807 to 008-CS0807 at the Herbarium of the National University of Singapore, Raffles Museum of Biodiversity Research and the Department of Biological Sciences of the National University of Singapore (Table 1).

#### Chemicals

2,2-diphenyl-1-picryl-hydrazyl (DPPH), magnolol, honokiol (99.9%) and quercetin were purchased from Sigma Aldrich (USA).

#### Solvents

Absolute ethanol (99.9%, Far East Distiller, Singapore) was diluted with water to produce 80% (v/v) solution of ethanol for extraction. De-ionized water was used for extraction (by boiling and maceration), reconstitution and dilution where appropriate. Methanol (analytical grade, Tedia, USA) was used for reconstitution and dilution in the DPPH assay.

#### Microorganisms, growth media and standard antibiotic discs

Four strains of bacteria and one strain of yeast were used for antimicrobial tests. The test bacteria included Gram-positive *Staphylococcus aureus *(ATCC 6538P) and *Bacillus subtilis *(ATCC 6633), Gram-negative *Pseudomonas aeruginosa *(ATCC 9027) and acid-fast *Mycobacterium smegmatis *(ATCC 14468). *Candida albicans *(ATCC 2091) was used as a representative of yeast. All microorganisms were purchased in the form of inoculation loops from Oxoid (UK). Nutrient broth with agar and Sabouraud dextrose agar (Acumedia, USA) were used for the cultivation of bacteria and yeast respectively. Mueller Hinton agar (France) was used in antimicrobial screening.

Standard antibiotic discs (diameter 6 mm) used in this study were: methicillin 5 μg, tetracycline 30 μg, carbenicillin 100 μg and streptomycin 10 μg. In our preliminary studies, these antibiotics were found to be active against *Staphylococcus aureus, Bacillus subtilis, Pseudomonas aeruginosa *and *Mycobacterium smegmatis *respectively. All standard antibiotic discs were purchased from Oxoid (UK). Disc containing chlorhexidine which was active against *Candida albicans*, were prepared by loading dry sterile filter paper discs (Whatman No. 54, diameter 5.5 mm) with chlorhexidine solution to give a total weight of approximately 100 μg of chlorhexidine per disc. The impregnated discs were dried overnight at 40°C and stored (less than five days) in a desiccator until use.

#### Preparation of plant materials prior to extraction

The fresh plants were kept in a refrigerator for no longer than three days prior to extraction. *Cortex Magnoliae Officinalis *was dried in a cool, dark room (room temperature 19°C, relative humidity 60%) and subsequently stored in a drum with silica gel desiccants until use. Before extraction, the plants were cut into 1 cm pieces with pruning scissors, except *Rhizoma Imperatae *and *Cortex Magnoliae Officinalis *which were milled into fine powder using a pulverizer mill (Christy & Norris, UK). Triplicate preparations of each sample were carried out.

### Extraction and preparation of crude extracts

#### Boiling, maceration and blending

Two and a half grams of *Folium Murraya Koenigii*, *Typhonium flagelliforme *aerial parts and 5 g of the other plant materials, were each extracted with 200 ml of water or ethanol. Three extraction methods were employed: (1) boiling in water for 1 hour, (2) maceration for 24 hours in water or (3) 80% (v/v) ethanol at room temperature. *Herba Houttuyniae *was extracted using an additional extraction method that involved boiling in water for 20 minutes [36]. Additional extraction experiments were carried out on aqueous plant extracts that showed promising antimicrobial activities. Boiling time was limited to 20 minutes to minimize heat exposure. Blending-maceration was used as a non-heat extraction method with cell rupture mechanism. Blending was performed with a laboratory blender (Waring Commercial, USA) for one minute, followed by a pause and then blending for an additional minute. Maceration in de-ionized water for one hour was performed. Coarse particles were removed using Whatman No. 1 filter paper (Whatman International, UK) before evaporation.

#### Extraction of fresh juices

Fresh juices of *Herba Houttuyniae*, *Epipremnum pinnatum *stem and *Typhonium flagelliforme *aerial parts and rhizomes were prepared in a mortar, wrapped in linen cloth and squeezed for the juices. Coarse particles were removed using Whatman No. 1 filter paper before evaporation.

#### Evaporation of extracts

The plant extracts were evaporated to dryness under reduced pressure at 40°C for ethanol extracts and 60°C for water extracts and fresh juices in a rotary evaporator (Model N1000, Eyela, Japan). The solid content of the extract was weighed. The dried extracts were stored in a freezer at -20°C.

#### Characterization of plant extracts

The crude and dried extracts were characterized by their odor, appearance and texture. The weights of the dried extracts were also determined.

### Determination of antimicrobial activities

#### Preparation of extract- and standard-loaded discs

Filter paper discs (Grade 54, diameter 5.5 mm, Whatman International, UK) were autoclaved at 121°C for 20 minutes and oven-dried at 40°C overnight. Plant extracts were diluted with the same extraction solvent to 50 μg/μl. Each diluted solution (2 μl, equivalent to 100 μg of the dried extract) was loaded on a sterile filter paper disc. All impregnated discs were dried in sterile glass Petri dishes placed in an oven at 40°C overnight. The discs were then allowed to condition to room temperature before use in the antimicrobial test. Solutions in methanol (5 μg/μl) were prepared for magnolol and honokiol respectively and a 1:1 solution of the two compounds (2.5 μg/μl) was made. 2 μl of the honokiol, magnolol or 1:1 solutions were loaded onto paper discs which were then left to air-dry. These standard-loaded discs were freshly prepared before the antimicrobial screening experiments.

#### Screening of antimicrobial activities of plant extracts

The antimicrobial activities of the extracts were determined by the Kirby-Bauer agar diffusion method according to NCCLS standards [37,38]. Sterilized molten agar (20 ml) was dispensed to each sterile disposable Petri dish (diameter 9 cm) and allowed to solidify. Mueller Hinton agar was used for bacteria and Sabouraud dextrose agar for yeast. Microbial suspension (200 μl) containing approximately 3 × 10^6 ^CFU was spread evenly onto the surface of the solidified medium. The plates were allowed to dry for 15 minutes before the test discs were placed at equidistance from each other. Each plate consisted of one standard antibiotic disc and three other discs impregnated with various extracts.

After standing for 30 minutes, the Petri dishes were incubated in an inverted position at 37°C for 18 to 24 hours for bacteria and 24°C for 48 to 72 hours for yeasts. The diameters of the zone of inhibition (ZIH), defined by the clear area devoid of growth, was measured twice. The antimicrobial activities were determined by the ratio of the ZIH diameters of the extracts to that of the standard antibiotic in the same Petri dish, whereby a higher ratio indicates a more potent extract.

#### Determination of antioxidant activity

Antioxidant activities of the extracts were determined with 2,2-diphenyl-1-picryl-hydrazyl (DPPH) assay [39]. The free radical, DPPH, served as the model oxidizing agent to be reduced by the antioxidant present in the extracts. The amount of dried extract subject to DPPH assay was 100 μg, the same amount used for antimicrobial screening. The dried extract was dissolved in 1.56 ml of methanol and mixed with 40 μl of 2 mM DPPH dissolved in methanol to make up a total volume of 1.6 ml in each polyethylene microfuge tubes. The final solution was allowed to react in dim light for 15 minutes. It was then centrifuged (4000 rpm; 1165 × *g*, Kubota 2100 Centrifuge, Japan) for five minutes. The absorbance of the supernatant was measured at 517 nm with a UV spectrophotometer (Genesys 10 UV, ThermoSprectronic, USA). The tests were carried out in triplicates. The DPPH radical scavenging activity was calculated with the following formula:

DPPH radical scavenging activity (%) = [A_0_-(A_1_-A_S_)]/A_0 _× 100

Where A_0 _is the absorbance of the control solution containing only DPPH after incubation; A_1 _is the absorbance in the presence of plant extract in DPPH solution after incubation; and A_s _is the absorbance of sample extract solution without DPPH for baseline correction arising from unequal color of the sample solutions (optical blank for A_1_).

#### Data and statistical analysis

Data are expressed as mean ± standard deviation (SD) of triplicates. Two-way ANOVA was used to analyze the effect of different plant materials and extraction methods on the extraction yields and DPPH radical scavenging activity while one-way ANOVA was performed to determine the effect of streptomycin, honokiol, magnolol and honokiol-magnolol combination on *M. smegmatis*. Both tests employed Bonferroni *post hoc *analysis. Student's t-test was used to compare antimicrobial activity of the extracts against the standard antibiotic. All statistical analyses were conducted with SPSS software (v.12, SPSS, USA) at a significance level of 0.05.

## Results and discussion

### Physical characterization of herbal extracts

#### Extraction yields

**Figure 1:**
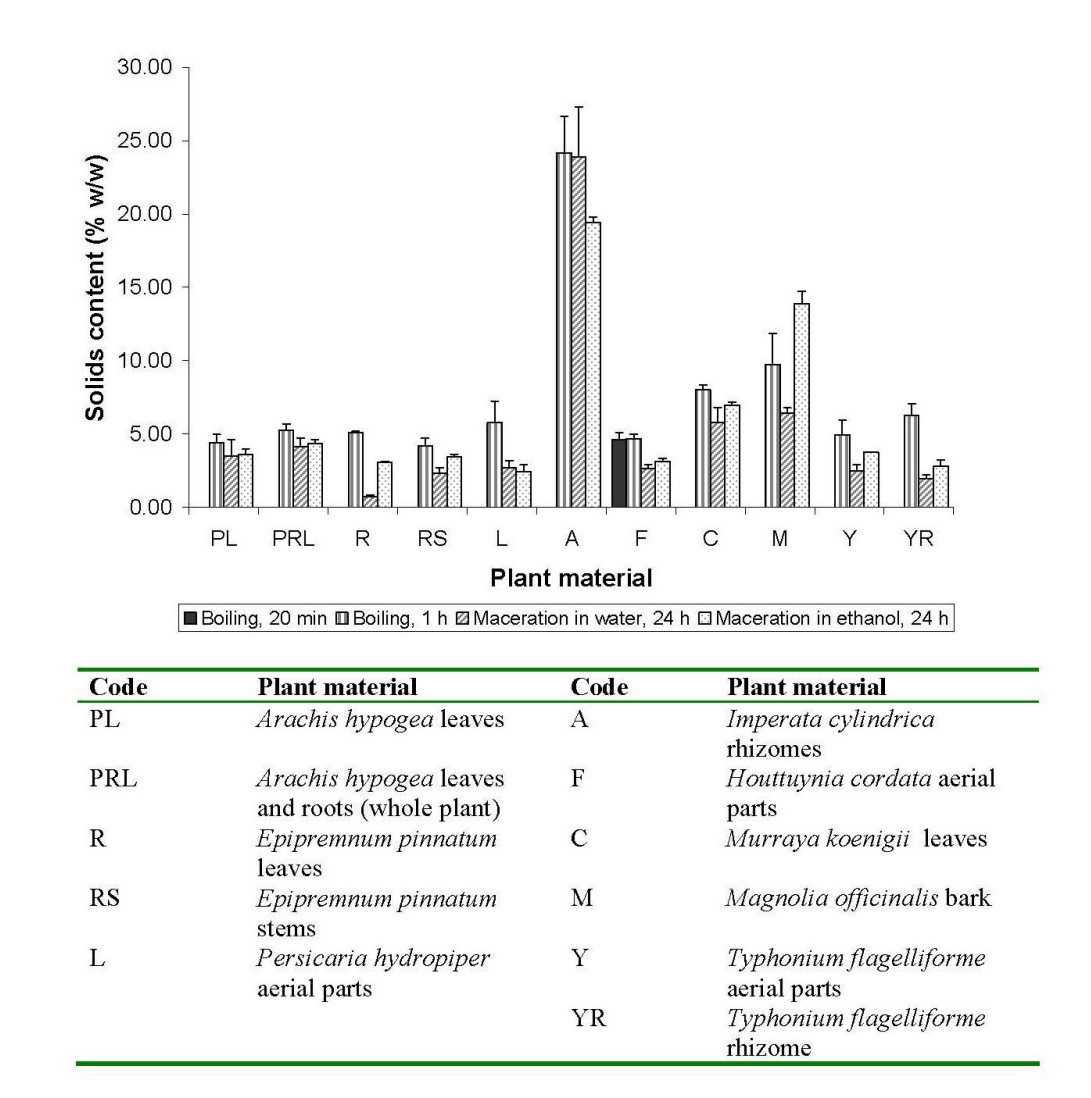
Solid content of extracts obtained by different methods*. *Error bars represent standard deviation (n = 3).

The extraction yields obtained from different extraction methods were analyzed with two-way ANOVA and Bonferroni *post hoc *analysis. Among the 11 experimental groups, *Rhizoma Imperatae *produced the highest yields (*P *= 0.001) regardless of extraction methods, followed by *Cortex Magnoliae Officinalis *(Figure 1). These two dry herbs were processed through comminution producing fine powder prior to extraction. The reduced particle size decreases the internal mass resistance for compounds to traverse through the plant matrix and increases the specific surface area for extraction. The extraction yields obtained from boiling were higher than those from other extraction methods.

Boiling *Herba Houttuyniae *aerial parts in water for 20 minutes or one hour produced comparable yields (*P *= 1.000). For *Herba Polygonis Hydropiperis*, *Folium Murraya Koenigii *and *Cortex Magnoliae Officinalis*, a shorter boiling time of 20 minutes was shown to be comparable to a boiling time of 60 minutes (*P *= 0.061, 0.053 and 0.798 respectively). While results from blending/maceration varied, this method was as efficient as the boiling method in terms of solid yields (*P *= 0.261) of *Folium murraya koenigii*.

#### Organoleptic properties

The color, texture and odor of the plant extracts were characterized (Additional file 1). The ethanolic extracts were better than corresponding aqueous extracts in retaining the natural fragrances of the plants. This may be due to the preservative ability of ethanol (i.e. reducing breakdown of organic compounds by microorganisms), its enhanced extraction capability (i.e. more fragrant components extracted) or a combination of both. Extracts obtained by boiling generally appeared darker and more turbid than those obtained by maceration. The solid content by boiling was higher than that by maceration (Figure 1). Boiling is more likely to damage the plant cell membrane and cell wall which act as natural filters to keep larger extraneous compounds within the cell.

### Antimicrobial activities

#### Dried herbal extracts

Among all the extracts studied, the 100 μg of the ethanolic extract of *Cortex Magnoliae Officinalis *loaded on the filter paper disc demonstrated the most robust antimicrobial activities against *S. aureus*, *B. subtilis*, *M*.*smegmatis *and *C. albicans*, equivalent to at least 50% of the activities of the standard antibiotics. Among the test organisms, it was most active against *M*.*smegmatis*, 20% more than the standard antibiotic, streptomycin 10 μg (Student's t-test, *P *= 0.001) (Table 2). The boiled extract of *Cortex Magnoliae Officinalis *had comparable antimicrobial activities to those of streptomycin 10 μg (Student's t-test, *P *= 0.279). These data suggest that *Cortex Magnoliae Officinalis *may be a potential agent to treat infections caused by *M. smegmatis *and *Mycobacterium tuberculosis *[40]. It was reported that magnolol and honokiol exhibited antibacterial activities against methicillin-resistant *S. aureus *and vancomycin-resistant enterococci [33], *Propionibacterium sp *[32] and periodontal pathogens [34]. Therefore, disk diffusion test was carried out on magnolol and honokiol individually and in combination (Table 2). The one way ANOVA on the four treatment groups namely streptomycin, honokiol, magnolol and combination of magnolol and honokiol (1:1) demonstrated a significant difference between groups (*P *= 0.001). Bonferroni *post-hoc *test showed that honokiol and magnolol had comparable activities (*P *= 1.000) against *M. smegmatis*, accounting for 83.58 ± 3.06% (*P *= 0.015) and 82.09 ± 6.51% (*P *= 0.006) of those of Streptomycin 10 μg respectively. In terms of antibacterial activities, the combination of magnolol and honokiol (1:1) was comparable to the reference antibiotic (*P *= 1.000) but higher than either magnolol (*P *= 0.007) or honokiol (*P *= 0.017) alone. These results suggest a new discovery of synergism between magnolol and honokiol.

**Table 2:** Inhibition zones of Streptomycin 10 μg, magnolol, honokiol and a 1:1 combination of magnolol and honokiol

**Compound tested**	**Inhibition zone (mm)**	**Percentage activity of compound in comparison to Streptomycin 10 μg (%)**	***P*-value** **(one-way ANOVA with Bonferroni *post hoc *test)**			
			Streptomycin 10 μg	Magnolol	Honokiol	Magnolol and Honokiol (1:1)
Streptomycin 10 μg	34.67 ± 1.75	100	-	-	-	-
Magnolol 10 μg	27.50 ± 2.18	82.09 ± 6.51	0.006	-	-	--
Honokiol 10 μg	28.42 ± 1.04	83.58 ± 3.06	0.015	1.000	-	-
Magnolol and Honokiol (1:1) 10 μg	34.50 ± 2.00	94.52 ± 5.48	1.000	0.007	0.017	-

Ethanolic extract of *Folium Murraya Koenigii *and boiled extract of *Herba Polygonis Hydropiperis *showed 80% and 50% of the activities of streptomycin 10 μg against *M. smegmatis *respectively. These extracts also exhibited antimicrobial activities against *S. aureus *and *B. subtilis*. Additionally, the boiled extract of *Herba Polygonis Hydropiperis *was active against *C. albicans*. Boiling was essential for the active principles to be removed from the laksa plant, as blended and water macerated extracts showed little antimicrobial activities (Table 3). The duration of the boiling process also affected the antimicrobial activities of laksa plant, whereby herbs boiled for 20 minutes were more active against *S. aureus *and *M. smegmatis*. The aerial parts of *Herba Houttuyniae *and rodent tuber were only active against *B. subtilis *and *S. aureus *respectively. The leaves and *Rhizoma Arachis Hypogea, Rhizoma Imperatae*, *Rhizoma Typhonium Flagelliforme*, and the leaves and stems *of Epipremnum pinnatum *did not show any antimicrobial activities.

**Table 3:** Antimicrobial activities of various plant extracts (100 μg of the extract per loaded disc)

			**Zone of inhibition (extract)/Zone of inhibition (standard)**									
			***S. aureus***		***B. subtilis***		***Ps. Aeruginosa***		***M. smegmatis***		***C. albicans***	
Plant name (Latin)	Plant part	Code	Ave	SD	Ave	SD	Ave	SD	Ave	SD	Ave	SD
*Arachis hypogea*	Leaves	PLB	-	-	-	-	-	-	-	-	-	-
		PLW	-	-	-	-	-	-	-	-	-	-
		PLE	-	-	-	-	-	-	-	-	-	-
	Rhizomes	PRLB	-	-	-	-	-	-	-	-	-	-
		PRLW	-	-	-	-	-	-	-	-	-	-
		PRLE	-	-	-	-	-	-	-	-	-	-
*Epipremnum pinnatum*	Leaves	RB	-	-	-	-	-	-	-	-	-	-
		RW	-	-	-	-	-	-	-	-	-	-
		RE	-	-	-	-	-	-	-	-	-	-
	Stems	RSB	-	-	-	-	-	-	-	-	-	-
		RSW	-	-	-	-	-	-	-	-	-	-
		RSE	-	-	-	-	-	-	-	-	-	-
	Stems (fresh juices)	RF	0.29	0.01	-	-	-	-	-	-	-	-
*Periscaria hydropiper*	Aerial parts (leaves & stems)	LB	0.30	0.02	0.32	0.02	-	-	0.50	0.00	0.50	0.00
		LW	-	-	-	-	-	-	-	-	-	-
		LE	0.25	0.02	-	-	-	-	-	-	-	-
		LM	-	-	-	-	-	-	-	-	-	-
*Imperata cylindrica*	Rhizomes	AB	-	-	-	-	-	-	-	-	-	-
		AW	-	-	-	-	-	-	-	-	-	-
		AE	-	-	-	-	-	-	-	-	-	-
*Houttuynia cordata*	Aerial parts (leaves & stems)	FSB	-	-	-	-	-	-	-	-	-	-
		FB	-	-	0.30	0.00	-	-	-	-	-	-
		FW	-	-	-	-	-	-	-	-	-	-
		FE	-	-	-	-	-	-	-	-	-	-
	Aerial parts (fresh juices)	FF	0.29	0.04	-	-	-	-	-	-	-	-
*Murraya koenigii*	Leaves	CB	-	-	0.30	0.00	-	-	-	-	-	-
		CW	-	-	-	-	-	-	-	-	-	-
		CE	0.49	0.01	0.35	0.03	-	-	0.77	0.05	-	-
		CM	0.20	0.02	-	-	-	-	-	-	-	-
*Magnolia officinalis*	Barks	MB	0.35	0.06	0.37	0.00	-	-	1.07	0.06	-	-
		MW	0.30	0.01	0.32	0.02	-	-	0.71	0.05	-	-
		ME	0.50	0.01	0.61	0.03	-	-	1.23	0.05	0.89	0.06
		MM	0.19	0.04	-	-	-	-	0.24	0.08	-	-
*Typhonium flagelliforme*	Aerial parts (leaves & stems)	YB	-	-	-	-	-	-	-	-	-	-
		YW	0.22	0.01	-	-	-	-	-	-	-	-
		YE	-	-	-	-	-	-	-	-	-	-
	Rhizomes	YRB	-	-	-	-	-	-	-	-	-	-
		YRW	-	-	-	-	-	-	-	-	-	-
		YRE	-	-	-	-	-	-	-	-	-	-
	Leaves (fresh juices)	YF	0.28	0.06	-	-	-	-	-	-	-	-
	Rhizomes (fresh juices)	YRF	0.27	0.02	-	-	-	-	-	-	-	-
	Roots (fresh juices)	YRR	0.23	0.02	-	-	-	-	-	-	-	-

An extract with a high yield, however, does not necessarily have high antimicrobial activities. For example, *Rhizoma Imperatae *whose yields topped all extraction methods, did not show any antimicrobial activities (Figure 1 and Table 3).

#### Fresh herbal extracts

The fresh juices of *Herba Houttuyniae *aerial parts, *Epipremnum pinnatum *stems and *Rhizoma Typhonium Flagelliforme *were tested for their folkloric use to treat wounds and various skin ailments (Table 1). All these fresh juices displayed some activities (less than 30% of the activity of methicillin 5 μg) against *S. aureus*. However, they were inactive against the rest of the test organisms. While the yields of fresh juices were lower than those of other extraction methods, antibacterial activities against *S. aureus *implied reduced degradation of the bioactive principles.

Among all extracts, only the fresh juices of *Rhizoma Typhonium Flagelliforme *and *Epipremnum pinnatum *leaves and stems possessed antimicrobial activities (Table 2), suggesting that the antimicrobial components were unstable and destroyed by boiling and/or maceration. However, the extract of aerial parts of *Rhizoma Typhonium Flagelliforme *by maceration in water showed 20% of the activities of methicillin 5 μg against *S. aureus*. This finding suggests the antimicrobial potential of rodent tuber is beyond the ethnomedicinal use of the rhizomes.

The strongly aromatic plant materials, such as *Herba Polygonis Hydropiperis*, *Folium Murraya Koeniggi *and *Cortex Magnoliae Officinalis*, exhibited a broad spectrum of antimicrobial activities. One possible reason is the presence of essential oils and active polyphenolic compounds which possess antimicrobial activities. Among the extracts of *Cortex Magnoliae Officinalis*, the ethanolic extract demonstrated the strongest activities against *S. aureus, B. subtilis*, *M. smegmatis *and *C. albicans*. The active biphenol compounds in *Cortex Magnoliae Officinalis *(honokiol and magnolol) are poorly water soluble and extracted more efficiently by ethanol than water.

None of the extracts, however, inhibited *Ps. aeruginosa*. Both *S. aureus *and *B. subtilis *are Gram-positive, while *Ps. aeruginosa *is Gram-negative and has an outer lipid membrane [41]. The results suggest that the antimicrobial compounds in the extracts were unable to penetrate this lipid membrane to exert their effects inside a cell. This speculation will require further experiments to confirm.

#### Antioxidant activities

**Figure 2:**
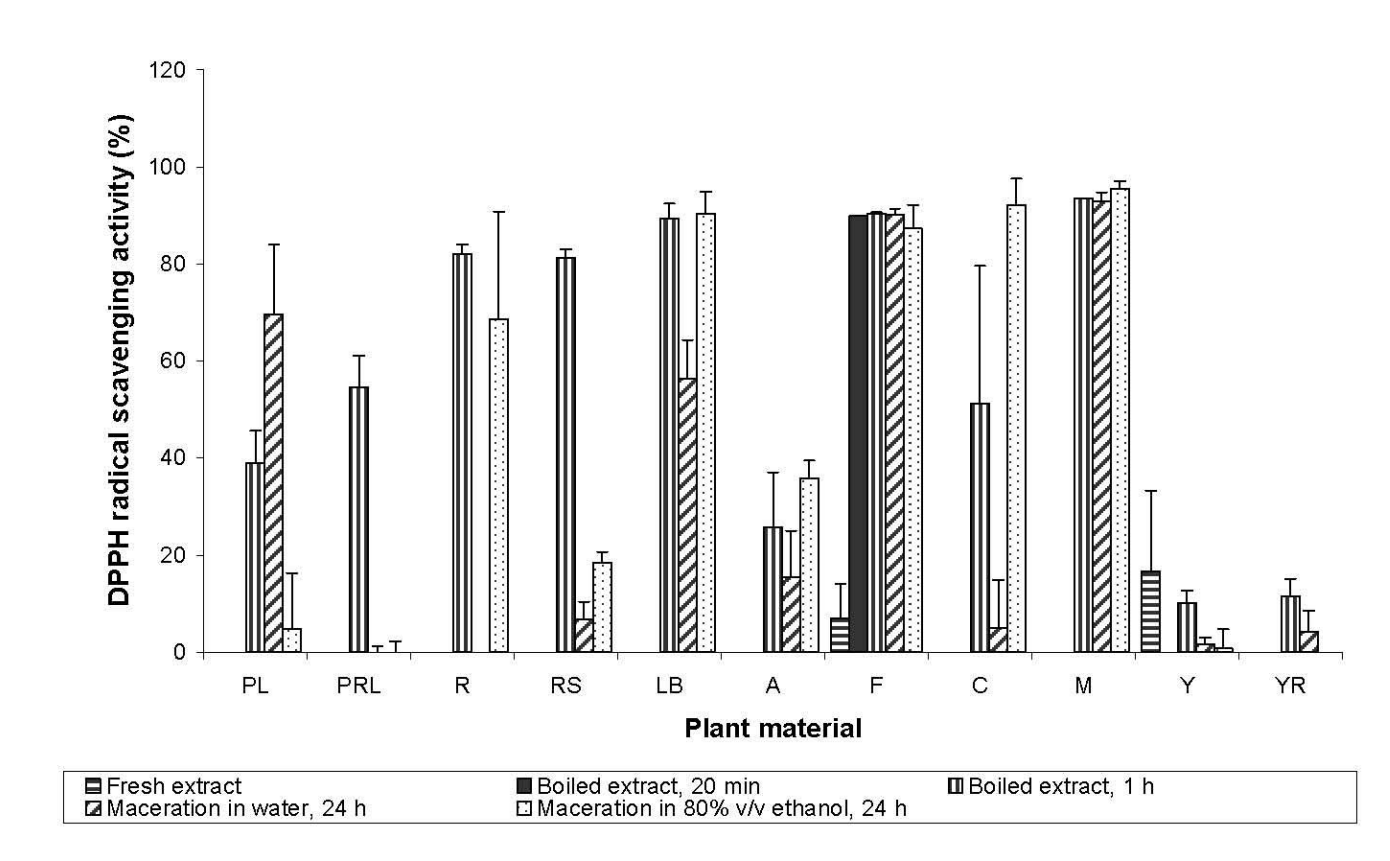
Antioxidant activities of extracts tested by DPPH assay*. *Error bars represent standard deviations (n = 3).

The antioxidant activities of the dried extracts and fresh juices are presented in Figure 2. All tested plants possessed some DPPH radical scavenging activities to a certain extent. While *Cortex Magnoliae Officinalis*, stems and leaves of dragon tail, laksa aerial parts, *Herba Houttuyniae *aerial parts and curry leaves showed high activities, rodent tuber rhizomes and aerial parts showed low activities.

The high antioxidant activities of the boiled and ethanolic extracts of the leafy materials were probably due to the extracted tannins and photosynthetic pigments. *Cortex Magnoliae Officinalis *is a rich source for antioxidative compounds, such as biphenols, polyphenols and tannins [42,43]. Lo *et al*. found that the antioxidant effects of magnolol and honokiol isolated from *Cortex Magnoliae Officinalis *were 1000 times higher than those of alpha-tocopherol [44]. Earlier studies confirmed that several naturally occurring dietary phytochemicals, such as isothiocyanates, curcumin and Epigallocatechin-3-gallate, possessed cancer preventive properties [45,46].

Boiled extracts showed greater antioxidant activities than those of other extraction methods (*P *= 0.001). Antioxidant compounds in leafy materials are generally located in conduit structures called the apoplast and symplast [47-49]. Maceration alone is not sufficient to extract these compounds from the structures. The application of heat, in the boiling process, facilitates cell rupture and leaching, thereby improving the mass transfer of these compounds from the storage organs into the boiling water. Ethanol may partially solubilize the membranes of the plant cells and storage organs, helping leach the chemicals away. However, maceration in 80% ethanol took over 24 hours and exposed the extracts to oxidative and hydrolytic degradation. This may explain the relatively low antioxidant activities of some ethanolic extracts.

The extracts of *Cortex Magnoliae Officinalis*, *Herba Houttuyniae *aerial parts and *Folium Murraya Koenigii *(ethanolic extract) had similar high DDPH radical scavenging activities (>85%) but markedly different antimicrobial properties (Figure 2 and Table 3). The results suggest that the active components for antimicrobial and antioxidant activities do not share common biochemical pathways.

## Conclusion

The present study discovered that (a) the ethanolic extract of *Cortex Magnoliae Officinalis *had 20% greater antimicrobial activities against *M. smegmatis *than streptomycin; (b) the boiled extract of *Cortex Magnoliae Officinalis *demonstrated comparable activities to streptomycin (c) the synergism of magnonol and honokiol had comparable effects to those of streptomycin; (d) the aerial parts of rodent tuber had antimicrobial activities against *S. aureus*. Among the tested 107 extracts, *Cortex Magnoliae Officinalis *had (1) potent antimicrobial activities against *S. aureus*, *B. subtilis*, *M. smegmatis *and *C. albicans *and (2) highest antioxidant activities in DPPH assay regardless extraction methods. *Cortex Magnoliae Officinalis *is likely a potential medicinal plant resource for developing effective antimicrobials and antioxidants.

## Competing interests

The authors declare that they have no competing interests.

## Authors' contributions

LWC and PWSH conceived the research design and supervised the manuscript preparation. WYW, ELCC and CLLS performed all extractions, antimicrobial and antioxidant studies as well as statistical analyses. All authors read and approved the final version of the manuscript.

## Supplementary Material

Additional file 1 Organoleptic properties of the plant extracts investigated in this study. This table is a summary of the organoleptic properties of the plant extracts investigated in this study, such as color, texture and odor. Click here for file
