# Supplementary material for: Antimicrobial and antioxidant activities of Cortex Magnoliae Officinalis and some other medicinal plants commonly used in South-East Asia
Source: Chin Med. 2008 Nov 28;3:15. doi: 10.1186/1749-8546-3-15 (PMC2631514; doi:10.1186/1749-8546-3-15)
Supplement: Additional file 1 — Organoleptic properties of the plant extracts investigated in this study. This table is a summary of the organoleptic properties of the plant extracts investigated in this study, such as color, texture and odor. [file 1749-8546-3-15-S1.doc]

**Additional file 1**

Organoleptic properties of plant extracts

| **Plant name (Latin)** | **Plant part** | **Method** | **Solvent** | **Code** | **Wet extract** | | | **Dried extract** | | |
| --- | --- | --- | --- | --- | --- | --- | --- | --- | --- | --- |
|  |  |  |  |  | **Color** | **Texture** | **Odor** | **Color** | **Texture** | **Odor** |
| *Arachis hypogea* | Aerial parts | Boil,1 h | Water | PLB | Murky, light brownish yellow | Slightly foamy when shaken | Sweet odor | Dark brown | Sticky, resinous | Sweet odor |
|  |  | Macerate, 24 h | Water | PLW | Murky, very light brownish yellow | Slightly foamy when shaken | Acrid | Brownish | Sticky, slightly waxy | Unpleasant smell |
|  |  | Macerate, 24 h | Ethanol | PLE | Clear, dark green | Water-like consistency | Alcoholic odor | Dark green | Waxy, thick | Leafy smell |
|  | Aerial parts and rhizomes (whole plant) | Boil, 1h | Water | PRLB | Less murky, very light yellow | Foamier than corresponding leaf & stem extracts | Sweet odor | Waxy, light brown | Waxy, sticky | Slight sweet odor |
|  |  | Macerate, 24 h | Water | PRLW | Murky, pale brownish yellow | Slightly foamy when shaken | Acrid, earthy smell | Golden brown | Waxy, sticky | Strong sweet smell, but trace of foul odor |
|  |  | Macerate, 24 h | Ethanol | PRLE | Light green | Water-like consistency | Alcoholic odor | Dark green | Shiny, waxy appearance | Leafy smell |
| *Epipremnum pinnatum* | Leaves | Boil, 1 h | Water | RB | Very murky, yellowish | Extremely foamy when shaken | Odor of seaweed | Dull greenish brown | Slimy | Unpleasant smelling |
|  |  | Macerate, 24 h | Water | RW | Clear, pale orange | Not foamy | Almost odorless (very slight sweetness) | Yellow-green | Powdery | Slight sweetness |
|  |  | Macerate, 24 h | Ethanol | RE | Clear, dark olive green | Liquid | Alcoholic odor | Dark green | Waxy/greasy, sticky | Slight sweet smell |
|  | Stems | Boil, 1 h | Water | RSB | Pale yellow- brown extract. Stems blackened after boiling | Viscous | Sweeter odor compared to leaf extracts | Dull brown | Sticky | Strong sweet smell |
|  |  | Macerate, 24 h | Water | RSW | Clear pale orange | Not foamy | Almost odorless (slight sweet smell | Murky beige | Flaky | Slight sweet smell |
|  |  | Macerate, 24 h | Ethanol | RSE | Clear deep golden yellow | Liquid | Alcoholic smell | Yellow- brown | Sticky, waxy (shiny) | Slight sweet smell |
| *Persicaria hydropiper* | Aerial parts (leaves and stems) | Boil, 1 h | Water | LB | Crude yellow | Not foamy | Characte-ristic odor of laksa plant | Dark brown | Slightly sticky | Smell of charring with hint of characteris-tic odor of laksa plant |
|  |  | Macerate, 24 h | Water | LW | Yellow, slightly murky | Not foamy | Characteris-tic odor of laksa plant but faint | Brownish | Flaky | Unpleasant smell |
|  |  | Macerate, 24 h | Ethanol | LE | Clear, bright green | Water-like consistency | Strong characteris-tic odor of laksa plant | Dark yellowish green | Waxy, slippery | Characteris-tic strong odor of laksa plant |
| *Imperata cylindrica* | Rhizomes (dried and milled) | Boil, 1 h | Water | AB | Yellowish | Not foamy | Smell of sugar cane | Yellowish brown | Sticky with caramel-like appearance and consistency | Strong sweet smell |
|  |  | Macerate, 24 h | Water | AW | Clear bright yellow | Water-like consistency | Sweet odor | Yellow | Sticky | Slight sweet smell |
|  |  | Macerate, 24 h | Ethanol | AE | Clear bright yellow | Water-like consistency | Alcoholic odor | Yellow, clear, glistening | Sticky | Smell of caramel |
| *Houttuynia cordata* | Leaves and stems | Boil, 20 min | Water | FSB | Crude yellow | Slightly foamy when shaken | Slightly pungent, fishy odor | Light golden brown | Sticky, clear | Sweet smell |
|  |  | Boil, 1 h | Water | FB | Crude yellow | Slightly foamy when shaken | Slight acrid | Golden brown | Sticky, clear | Acrid |
|  |  | Macerate, 24 h | Water | FW | Clear bright yellow | Not foamy | Fishy odor | Light yellow | Sticky | Faint sweet smell |
|  |  | Macerate, 24 h | Ethanol | FE | Clear, olive green | Water-like consistency | Slight fishy odor | Yellowish to greenish brown | Sticky but clear | Slight fishy but sweet odor |
| *Murraya koenigii* | Leaves | Boil, 1 h | Water | CB | Yellowish brown, murky | Slightly foamy when shaken | Odor of curry leaves, slightly pungent | Dark brown | Sticky | Faint sweetness |
|  |  | Macerate, 24 h | Water | CW | Clear, golden yellow | Not foamy | Typical odor of curry leaves | Dark brown | Sticky | Spicy odor of curry leaves |
|  |  | Macerate, 24 h | Ethanol | CE | Clear, very dark green | Water-like consistency | Alcoholic odor | Dark green (almost black) | Greasy / waxy | Faint odor of curry leaves, leafy smell |
| *Magnolia officinalis* | Barks (dried and milled) | Boil, 1 h | Water | MB | Dark reddish brown | Foamy with sediments | Strong magnolia bark smell, pleasant | Brown | Sticky, shiny | Magnolia bark smell |
|  |  | Macerate, 24 h | Water | MW | Golden yellowish brown | Slightly foamy | Slight magnolia bark smell, slightly pungent | Amber | Smooth surface | Magnolia bark smell |
|  |  | Macerate, 24 h | Ethanol | ME | Golden yellow-orange hue | Water-like consistency | Magnolia bark smell | Amber | Sticky, shiny | Magnolia bark smell |
| *Typhonium flagelliforme* | Aerial parts | Boil, 1 h | Water | YB | Clear, light greenish yellow | Slightly foamy on agitation | Almost odorless, slightly bitter | Yellow | Starchy | Slightly pungent |
|  |  | Macerate, 24 h | Water | YW | Murky, light yellow | Very foamy on agitation, slightly viscous | Leafy smell, a little pungent | Pale yellow, off white | Starchy | Strong pungent smell, faint sweet smell |
|  |  | Macerate, 24 h | Ethanol | YE | Clear yellowish green | Not foamy | Leafy smell | Green | Extracts with oily droplets | Sweet |
|  | Rhizomes | Boil, 1 h | Water | YRB | Murky, off-white | Thick, starchy, extremely foamy | Strong smell : Pungent and sweet | Off-white | Thick, starchy | Strong smell, sweet |
|  |  | Macerate, 24 h | Water | YRW | Murky, off-white | Thick, starchy, foamy | Pungent, sourish smell | Off white | Thick, starchy | Strong pungent smell |
|  |  | Macerate, 24 h | Ethanol | YRE | Slight murky, white | Not foamy | Slightly pungent smell | Pale yellow | Oily | Odorless |
